# CHALLENGES AND STRATEGIES FOR SUSTAINING A NEW DEMENTIA BEHAVIORAL CARE APPROACH IN NURSING HOMES

**DOI:** 10.1093/geroni/igz038.1393

**Published:** 2019-11-08

**Authors:** Michele J Karel, Karen M Benson, Youliana Piscopo, Susan Maataoui, Kimberly Curyto

**Affiliations:** 1 Office of Mental Health and Suicide Prevention, Veterans Health Administration, Washington, District of Columbia, United States; 2 Michael E. DeBakey VA Medical Center, Houston, Texas, United States; 3 John D Dingell VA Medical Center, Detroit, Michigan, United States; 4 VA Maine Healthcare System, Augusta, Maine, United States; 5 Batavia VA Medical Center, Batavia, New York, United States

## Abstract

The STAR-VA training program in Veterans Health Administration Community Living Centers (CLCs) has been helping interdisciplinary care teams understand and manage dementia-related behaviors in the nursing home setting, with promising clinical outcomes. However, sustaining a new care approach in a health care system poses multiple challenges. This presentation will discuss facilitators and barriers to STAR-VA sustainability based on CLC team and nurse leader feedback. Findings are informing development of a new site coaching program and a sustainability toolkit. Feedback to date suggests that critical STAR-VA implementation and sustainability strategies include: regularly scheduled team meetings to discuss behavioral assessment and care plans; ongoing staff training (e.g., new staff orientation); communicating care plans across shifts and in the health record; multiple nurse/shift champions; impromptu huddles; acknowledging staff successes; leadership engagement. The coaching program engages teams in setting and tracking site sustainability goals. Lessons learned will be discussed.

